# Macroepidemiologic assessment of swine disease co-occurrences in the United States of America

**DOI:** 10.1186/s40813-026-00487-4

**Published:** 2026-02-05

**Authors:** Guilherme A. Cezar, Eric R. Burrough, Rodger G. Main, Melanie Prarat Koscielny, Ryan Farmer, Ryan Yanez, Rafael R. Nicolino, Daniel C. L. Linhares, Giovani Trevisan

**Affiliations:** 1 Department of Veterinary Diagnostic and Production Animal Medicine, Iowa State University, Ames, IA USA; 2 Ohio Animal Disease Diagnostic Laboratory, Reynoldsburg, Ohio, USA; 3 Department of Preventive Veterinary Medicine, School of Veterinary Medicine, Federal University of Minas Gerais, Minas Gerais, Belo Horizonte, Brazil

**Keywords:** Co-infections, Porcine, Etiology, Pathogens, Viral, Bacterial

## Abstract

Swine producers frequently encounter polymicrobial disease challenges, with co-infections exacerbating clinical disease and complicating response strategies. This study aimed to characterize co-diagnosis patterns in swine by integrating confirmed diagnosis cases using a standardize diagnosis system (Dx code) from the Iowa State University Veterinary Diagnostic Laboratory. As a secondary objective, the study developed the concept and implemented a framework for the technological transfer of the Dx code system from ISU-VDL to the Ohio Animal Disease Diagnostic Laboratory, and to gather Dx code data through an animal disease monitoring program, thereby creating a multi-institutional, confirmed tissue disease diagnosis database. The final collated database was harmonized and used to analyze 45,310 confirmed tissue diagnosis cases submitted between 2020 and 2025. Co-diagnosis was defined as the presence of two or more distinct etiologies within a single case. Overall, 52.62% of cases were co-diagnosed in 42 U.S. states, with a seasonal variation indicating reduced submissions and co-diagnosis rates during the summer months. The wean-to-market production phase accounted for 86.45% of co-diagnosed cases. The co-diagnosed cases were more abundant in respiratory and systemic anatomic systems, with porcine reproductive and respiratory syndrome virus (PRRSV) and *Streptococcus suis* being the predominant co-diagnosed pathogens. Age-specific trends revealed respiratory co-diagnoses peaking in nursery and grow-finish pigs, while digestive co-diagnoses were more common in suckling piglets. Statistical modeling using Conway-Maxwell-Poisson regression revealed that co-diagnosis cases involving bacterial and viral insults had significantly higher numbers of distinct etiologies (IRR = 2.48; CI: 1.66–3.66) compared with co-diagnosis cases without bacterial or viral involvement, with an expected count of 2.95 distinct etiologies per case. The study demonstrated the value of standardized diagnostic coding for epidemiological surveillance and highlights the complexity of co-infections in swine. Additionally, the findings underscore the importance of collaborative data sharing in enhancing swine health management strategies.

## Introduction

Swine producers often encounter health challenges due to the presence of multiple pathogens. These co-infections are common in respiratory and enteric diseases and often complicate efforts to control them. In respiratory cases, the term “porcine respiratory disease complex” (PRDC) is used to describe the multifactorial nature of the condition, including porcine reproductive and respiratory syndrome (PRRSV), influenza A virus, porcine circovirus 2 (PCV2), *Streptococcus suis*, *Glaesserella parasuis*, and *Actinobacillus pleuropneumoniae* [1, 2]. Similarly, pig enteric diseases are frequently associated with several pathogens, including porcine enteric coronaviruses, *Escherichia coli*, *Salmonella*, and others [3]. The presence of multiple pathogens can lead to more severe clinical signs and poorer performance, even in pigs that appear clinically healthy [4].

Field studies have shown that co-infections are frequent and diverse, which makes diagnosis and control more difficult [5]. For example, in Eastern China, researchers found that over half of the pigs sampled carried more than one respiratory pathogen, with PCV2 and *Streptococcus suis* being the most common combination [6]. Economic analyses have estimated that co-infections can cost producers up to 30 euros per pig, depending on the pathogens involved and the severity of the disease [7]. In addition, co-infections led to exacerbation of other pathogens, as described between PRRSV and *Streptococcus suis* [8]. In enteric disease investigations, porcine enteric coronaviruses such as porcine epidemic diarrhea virus (PEDV) and porcine deltacoronavirus (PDCoV) are commonly investigated, especially in newborns, where mortality can be high [9]. These viruses are genetically distinct, which means immunity to one doesn’t protect against the other [10].

Despite their importance, co-infections are often challenging to detect, diagnose, and interpret. Diagnostic protocols are often designed to identify single pathogens, and co-detection data may be overlooked or underreported [11]. Although advances in molecular multiplex technologies have enhanced the ability to detect multiple targets in a single sample [11, 12], there is still a challenge in detecting multiple pathogens affecting animals in population medicine.

The Iowa State University Veterinary Diagnostic Laboratory (ISU-VDL) diagnostic coding (Dx code) system offers a structured approach to summarizing diagnoses in veterinary medicine [13]. Each code includes four elements—body system, type of insult, lesion or tissue affected, and the suspected or confirmed cause—allowing diagnosticians to consistently capture the essence of a case based on clinical history, pathology, and lab results [13]. The coding system integrates findings from the complete diagnostic workflow, including virus and bacterial cultures, molecular assays, serology, and histopathology, before a diagnostician assigns a final code (depending on the availability of the diagnostic test performed in the case). The Dx code approach increases diagnostic specificity at the case level compared to relying solely on the analysis of a single test result. The framework developed supports both retrospective and real-time analysis of disease patterns, particularly in swine populations, by enabling the aggregation of diagnostic data across time and geography [14]. Overall, the Dx code methodology enhances the epidemiological value of diagnostic data, supporting more informed decisions in animal health management. However, the current database includes only one VDL, and the addition of multiple VDLs could improve database representativeness, allowing for a better understanding of swine co-diagnosis trends, as demonstrated in similar initiatives that aggregates PCR diagnostic data [15, 16].

Therefore, this study focused on exploring the co-diagnosis patterns of swine etiologies and how these patterns shift by anatomic system, age category, and geographical location. Generated co-diagnosis trends were further used to offer valuable insights into the dynamics of swine disease and support more effective health management strategies in the field. A secondary objective was to develop the concept and implement a framework for the technological transfer of the Dx code system from one VDL to another, and to gather Dx code data through an animal disease monitoring program, thereby creating a multi-institutional, confirmed tissue disease diagnosis database.

## Materials and methods

### Data collection and handling

This study utilized confirmed diagnostic data from porcine cases that had tissue submitted from two veterinary diagnostic laboratories: ISU-VDL and Ohio’s Animal Disease Diagnostic Laboratory (OH-ADDL). The diagnostic code methodology developed by ISU-VDL was transferred to the OH-ADDL and implemented in their laboratory information management system (LIMS) to ensure consistency in diagnostic terminology. Usage of a standardized coding system enhances the accuracy of data aggregation across laboratories by harmonizing case results. In alignment with previously established methodologies [14], anonymized confirmed diagnosis submission records, including the four levels of the code (system, insult type, lesion type, and etiology), were collected, cleaned, and standardized at the submission level. All identifying information related to laboratory clients, including farm owners, veterinarians, and submitters, was anonymized before being integrated into the database. Initially, data collation was performed using a SAS script (SAS Version 9.4, SAS Institute Inc., Cary, NC). It was later transitioned to a web-based application developed in C# 10 using the.NET 6 framework. The processed data were securely stored in a Microsoft SQL Server database hosted by the Department of Veterinary Diagnostic and Production Animal Medicine (VDPAM) at Iowa State University. Each participating laboratory supplied a unique application programming interface (API) key, which enabled secure and automated daily retrieval of diagnostic data through “GET” calls. The system ensures that the central database remains consistently updated with the most recent submissions from the VDLs. Final datasets for confirmed diagnosis results were analyzed to identify detection trends over time from 2020 – 2025, considering variables such as systems affected, insult type affecting the case, lesions, age category, and the geographic origin of the samples.

### Disease diagnosis code collation

Diagnostic codes were structured at the submission level using the accession ID assigned by each VDL as a unique case identifier. In this context, a “case” refers to all information linked to a single accession ID. The date the samples were received, as reported by the VDLs, was used to determine the timeline. The site state listed on the VDL submission form served as the geographic reference. Records associated with research facilities, non-swine species, or samples originating outside the U.S. (e.g., Canada, Mexico) were excluded.

Confirmed tissue diagnosis cases from 2020 to 2025 were compiled using their respective accession IDs. Seasonal classification was derived from the case received date, with months grouped as follows: summer (June–August), fall (September–November), winter (December–February), and spring (March–May). Each annual cycle began on December 1 and ended on November 30 of the following year. Age categories were assigned based on farm type, age unit, and age data provided in the submissions. If farm type was available, it guided the classification (e.g., suckling piglets, nursery, grow-finish, breeding herd, replacement, boar stud). In cases lacking farm type, age ranges were used: 0–22 days (suckling piglets), 23–63 days (nursery), 64–200 days (grow-finish), and over 200 days (adults). These categories were then grouped into broader production types: adult/sow farm (including breeding herds, replacements, boar studs, suckling piglets, and adults) and wean-to-market (nursery-age and grow/finish-age). Submissions missing farm type and age-related data were labeled as “unknown.”

### Co-diagnosis framework and statistical analysis

The final collated dataset was structured to investigate co-diagnosis patterns in swine tissue submissions using ISU-VDL data only. OH-ADDL data were not included in this analysis because the implementation and collection of Dx codes at the laboratory occurred in 2025; therefore, no historical data were available. The dataset, initially organized by accession ID and diagnostic codes, was refined to exclude non-disease-related entries such as trauma, neoplasia, and unspecified abnormalities. Each accession ID was then evaluated for the presence of multiple distinct etiologies, and a binary indicator was assigned to identify co-diagnosed cases, defined as those with two or more distinct etiological agents within a case.

Further classification of cases was performed based on the type of insult, resulting in two columns indicating whether the case was affected or not by at least a virus or bacterium, creating columns labeled “Bacterial” and “Viral” as a binary outcome (1 = presence and 0 = absence). For example, a co-diagnosis case involving *Escherichia coli* and coccidiosis would be considered a case affected by a bacterium, which would have the binary outcomes “bacterial = 1” and “viral = 0” as it presents the presence of a bacterium and the absence of any viral insult.

Generalized linear models were employed to model the relationship between insult type and the number of distinct etiologies per co-diagnosis cases. The primary response variable was the distinct count of etiologies per accession ID. A Conway-Maxwell Poisson (COM-Poisson) regression was implemented, based on model fit was assessed using the Akaike Information Criterion (AIC) and root mean square error (RMSE). The dispersion statistic was computed by summing the squared Pearson residuals and dividing by the model’s residual degrees of freedom. A value close to 1 was considered that the model fits the data well under the Poisson assumption, while values below 1 suggested underdispersion. The expected count of etiologies per co-diagnosed case *i*, denoted by *λi*, was modeled as a log-linear function of insult type:

$$\lambda i = exp\left( {{{\rm{\beta }}_0} + {{\rm{\beta }}_1}{{\rm{X}}_{{\rm{i}}1}} + {{\rm{\beta }}_2}{{\rm{X}}_{{\rm{i}}2}} + {{\rm{\beta }}_3}{{\rm{X}}_{{\rm{i}}1}}{{\rm{X}}_{{\rm{i}}2}}} \right) $$

Where X_i1_ and X_i2_ are binary indicators for the presence of bacterial and viral insults, respectively, and X_i1_X_i2_ represents their interaction. The response variable Y_i_, corresponding to the number of distinct etiologies, follows a COM-Poisson distribution with *ν* being the dispersion parameter:

$${Y_i}{\rm{ }} \sim {\rm{ CMP }}\left( {{{\rm{\lambda }}_i},{\rm{ }}\nu } \right){\rm{ }} $$

Furthermore, the presence of PRRSV and *Streptococcus suis*, the most prevalent viral and bacterial pathogens identified in co-diagnosis cases of swine, was incorporated into the final model as covariates to assess their confounding effects.

### Analysis of trends over time

Access to the finalized dataset was facilitated using R (version 4.2.3; R Core Team), which connected directly to the centralized database and enabled local data processing. The dataset was integrated into Microsoft Power BI (Microsoft Corporation, Redmond, WA) for visualization purposes, providing an intuitive platform for exploring data trends. The refined framework was used to quantify co-diagnosis rates over time, by season, age category, and anatomical system. Visualizations such as filled maps, line graphs, and clustered column charts were employed to examine temporal and co-diagnosis patterns in the confirmed tissue diagnosis dataset.

## Results

A total of 58,170 cases with tissues were recorded in the confirmed diagnosis database between January 2020 and September 2025. After removing 12,860 cases that did not meet the eligibility criteria described in this study methodology, the total number of cases analyzed was 45,310. From the total number of cases, 30.12% (13,649 of 45,310) had at least one bacterium involved but no viruses (bacterial = 1 and viral = 0), where 30.15% (13,663 of 45,310) had at least one virus involved but no bacteria (bacterial = 0 and viral = 1). Additionally, 36.62% (16,595 of 45,310) of cases involved both bacterial and viral insults (viral = 1 and bacterial = 1), and the remaining 3.11% (1,406 of 45,310) of cases did not have either viruses or bacteria. The Dx code system was successfully implemented, and integration of the two laboratories (ISU-VDL and OH-ADDL) as data providers to the animal health monitoring program was completed in the fourth quarter of 2025. A total of 12 cases have been successfully integrated, coming from sites located in two states, Ohio and West Virginia, demonstrating the feasibility of using a standardized approach to register and retrieve confirmed disease Dx codes daily.

### Assessment of co-diagnosis cases over time

Out of the total 45,310 cases in the final database, 23,846 were categorized as co-diagnosis cases, with a range of 2 to 9 distinct etiologies within the same case. Therefore, considering the assessed data period, 52.62% of the confirmed tissue diagnosis cases had a co-diagnosis.

**Fig. 1:**
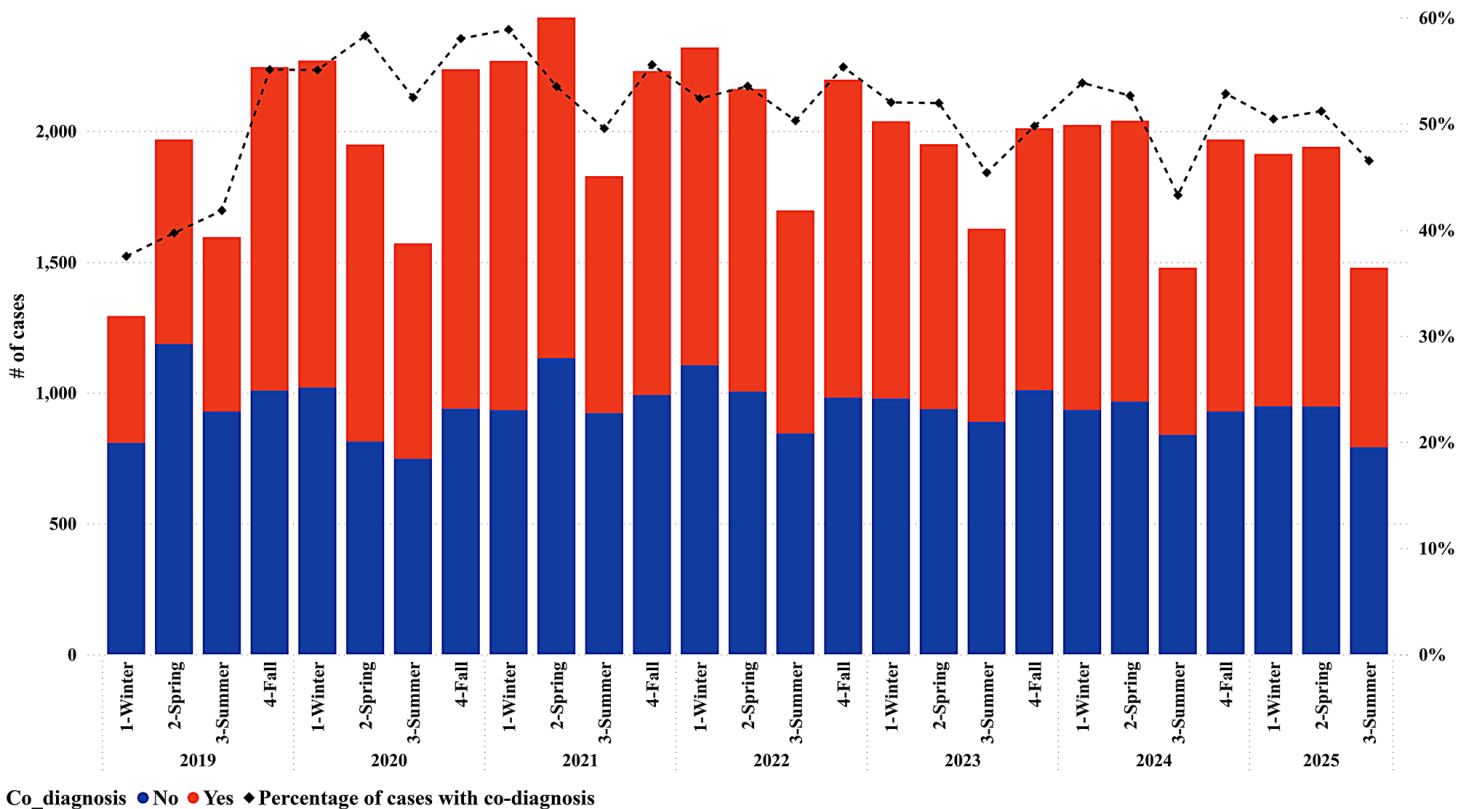
Total number of confirmed diagnosis cases by year and season. Each point (x-axis) represents a season within a year. The bars represent the cases tested and are color-coded by co-diagnosis results. The red color indicates cases with at least two distinct etiologies (co-diagnosis), and blue represents cases with a single etiology. The black line represents the percentage of co-diagnosis (secondary Y-axis)

A seasonal trend was identified, characterized by a decrease in the number of confirmed diagnosis submissions every summer and a minor reduction in the percentage rate of co-diagnosis cases (47.50%), compared with the average co-diagnosis rate of the database (Fig. 1). The highest percentage of co-diagnosis rate was recorded in winter 2021 (59%), which also had the highest absolute number of co-diagnosis cases in a single season (1,335). Additionally, 2021 saw the highest number of confirmed tissue diagnosis cases submitted in the Spring of that year (2,434).

Among the pig production phases, the wean-to-market stage accounted for the largest share of confirmed tissue diagnosis cases, representing 77.05% (34,914 out of 45,310 cases). In addition, the proportion of wean-to-market cases increased when we considered only the co-diagnosis cases, reaching 86.45% (20,616 out of 23,846 cases). On the other hand, the “unknown” production type had the lowest proportion, representing 6.28% of the cases. In 2025, the “unknown” production type achieved the lowest percentage of cases (4.58%), which may indicate improvements made by the submitter in providing this information during submission and advancements in VDLs’ data collection practices over time, such as the use of an electronic submission form. Finally, the adult/sow farm represented 16.67% of the cases (7,557 out of 45,310), demonstrating a marked difference between the wean-to-market production type and the others.

**Fig. 2:**
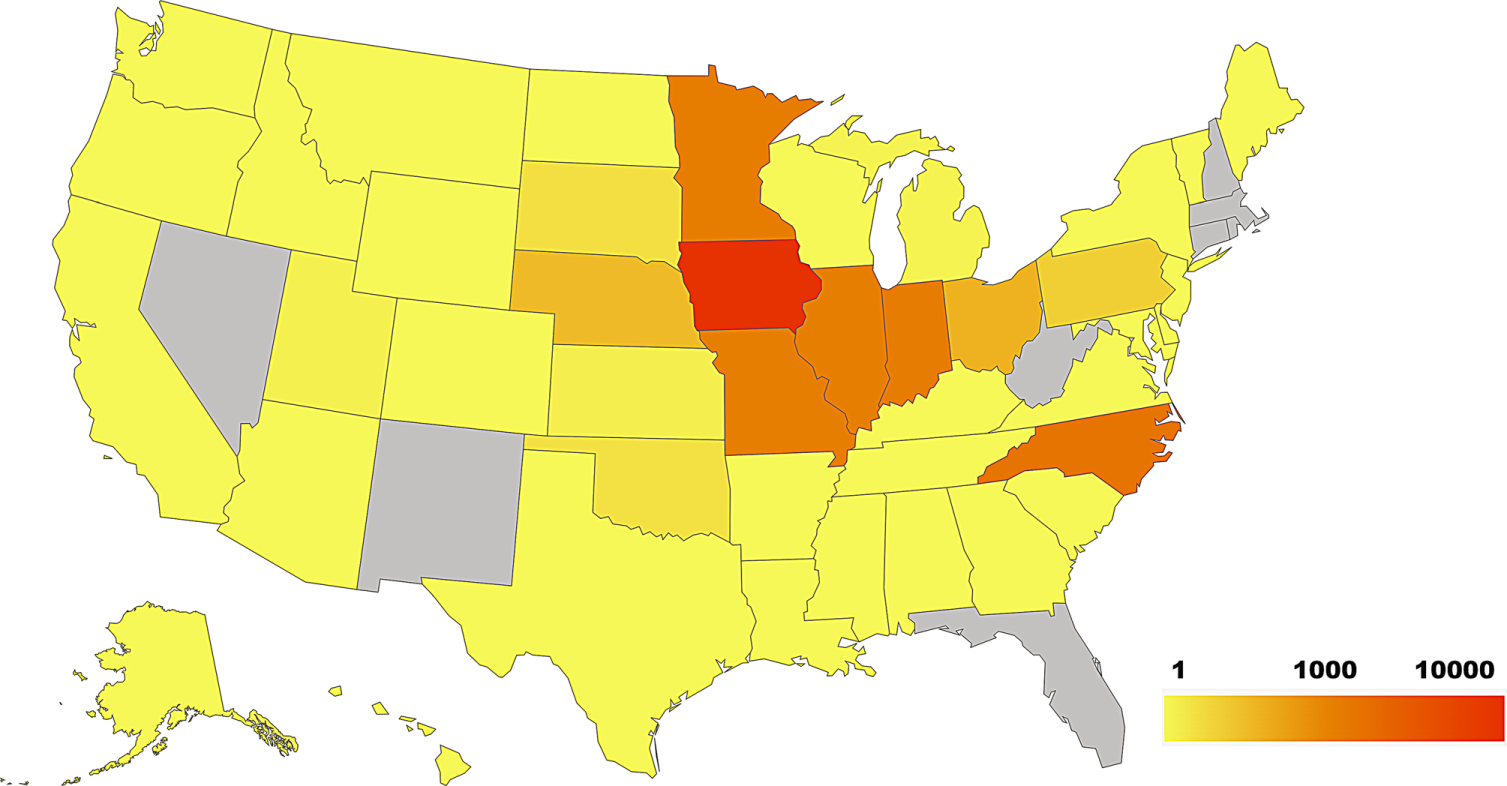
Distribution of the co-diagnosis cases by state in the United States from 2020 to 2025. Shades of yellow represent States with cases in between 1 and 1000, orange represents States with more than 1000, and red color States with more than 10,000 cases

Focusing on the co-diagnosis cases, geographically, they were submitted from 42 out of the 50 U.S. states, demonstrating a broad geographical representation (Fig. 2). Iowa concentrated most of the cases, with 13,332 of the 23,846 co-diagnosis cases. The U.S. Midwest region accounted for the majority of other co-diagnosis cases, with the largest numbers originating from Indiana (1,394), Minnesota (1,260), Missouri (1,134), and Illinois (1,023). The exception was North Carolina, located on the East Coast, which had the second-largest number of co-diagnosis cases in the database (2,231). These six states, with the highest number of co-diagnosis cases, represented 85.25% of the total number of co-diagnosis cases.

**Fig. 3:**
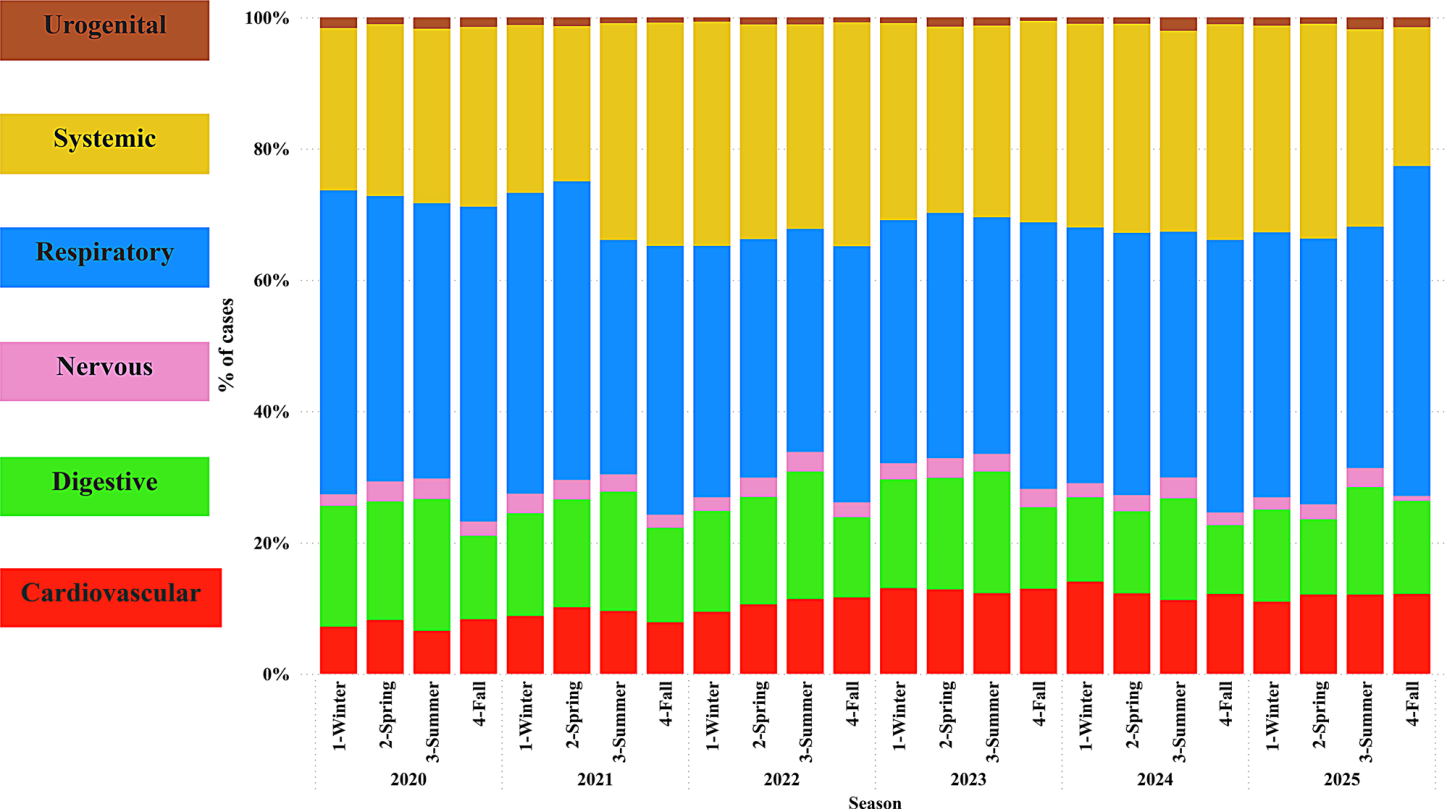
Proportion of co-diagnosis cases by anatomic systems by year and season. Each point (x-axis) represents a season within a year. Each color represents one anatomic system affected by the co-diagnosis cases: cardiovascular (red), digestive (green), nervous (pink), respiratory (blue), systemic (yellow), and urogenital (brown)

Regarding the animal body systems affected by these co-diagnosis cases, the respiratory system was the most commonly affected, accounting for 81.77% of the co-diagnosis cases (19,501 out of 23,846) that had at least one etiology affecting the respiratory system. The systemic system was involved in 61.35% of cases (14,631 out of 23,846), followed by the digestive system with 30.88% (7,365 out of 23,846), the cardiovascular system with 21.84% (5,210 out of 23,846), and the nervous system with 5.10% (1,216 out of 23,846), completing the body systems most affected by the etiologies. In addition, comparing the proportion of cases by season, the digestive system showed a 3.6% increase in cases in summer compared with the other seasons, while respiratory cases increased by 2.9% in fall compared with the other seasons (Fig. 3).

**Fig. 4:**
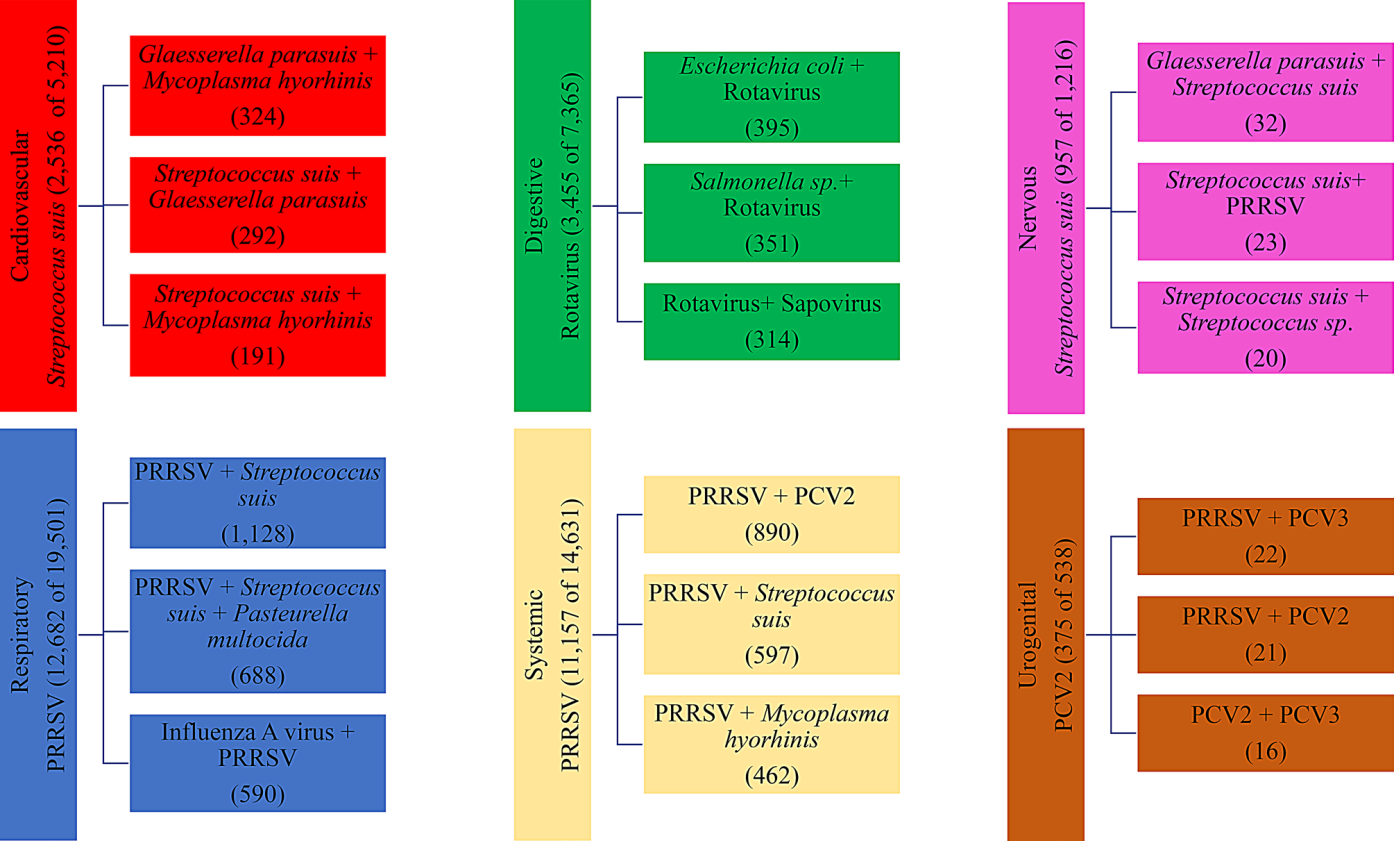
The most frequently co-diagnosed etiology by system, along with the number of co-diagnosis cases out of the total number of cases of this etiology within the system. Additionally, the three most frequently occurring combinations of etiologies within the anatomic system, along with the respective number of cases

It is essential to highlight that cases can involve tissues from multiple animals, which does not necessarily mean that all the etiologies found affecting a system originated from the same animal. Additionally, a case may have had affect multiple anatomic systems affected, which explains why the sum of all systems exceeded the total number of co-diagnosis cases (23,846). PRRSV and *Streptococcus suis* led the number of co-diagnosis cases involving multiple systems, being detected as the most frequently diagnosed pathogens in 4 out of the 5 systems most affected (cardiovascular, respiratory, nervous, and systemic) (Fig. 4). In addition, the most prevalent combinations within each system typically involved a combination of a virus and a bacterium, highlighting the interaction between these two types of insults.

**Fig. 5:**
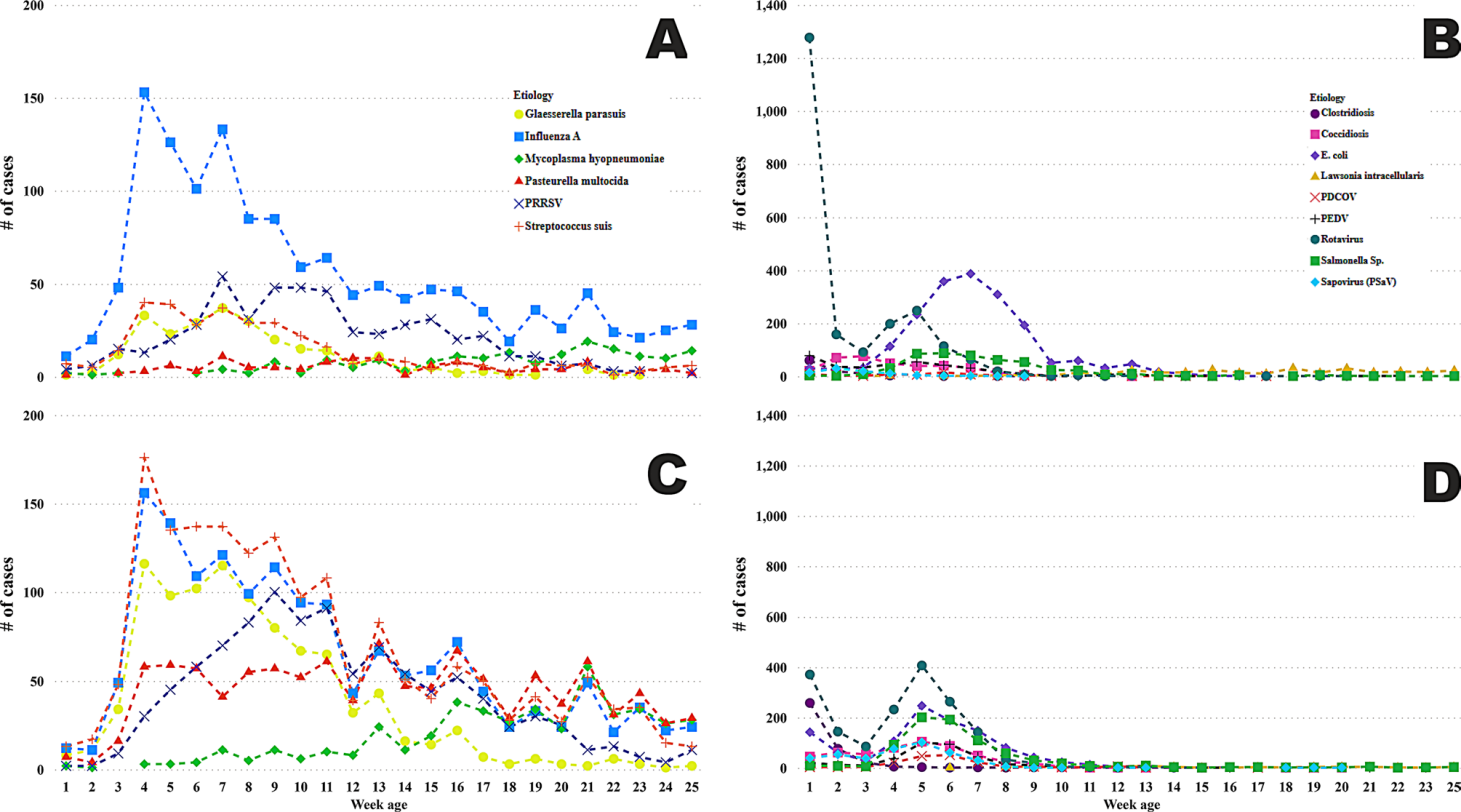
Number of confirmed diagnosis cases involving selected etiologies by anatomical system and animal age (in weeks) at the time of sample submission. Each point represents the number of confirmed cases for a specific etiology at a given age. Co-diagnosis refers to instances where the etiology was detected alongside at least one other distinct etiological agent. Each line color represents a different pathogen within each panel. Respiratory system within blue borders: (**A**) Distribution of respiratory cases attributed to a single etiology; (**C**) Distribution of respiratory cases having at least one additional distinct etiology co-diagnosed. Digestive system cases within green borders: (**B**) Distribution of digestive cases attributed to a single etiology; (**D**) Distribution of digestive cases with co-diagnosis. Porcine reproductive and respiratory system (PRRSV); porcine epidemic diarrhea virus (PEDV); porcine deltacoronavirus (PDCoV)

The number of cases by animal age in weeks varied by anatomic system, particularly between the digestive and respiratory systems. The respiratory diagnosis cases involving a single etiology occurred mainly after 3 weeks of age, which would be categorized as nursery animals, and continued with a high number of cases throughout the grow-finish phase of the animals (greater than 10 weeks of age). More than 90% of the cases involved PRRSV, influenza A virus (IAV), *Mycoplasma hyopneumoniae*, *Pasteurella multocida*, *Glaesserella parasuis*, or *Streptococcus suis* (Fig. 5A). The primary difference in the pattern of respiratory co-diagnosis cases was the increase in the number of bacterial cases, indicating the frequency with which respiratory bacterial pathogens co-occurred rather than as a single etiology (Fig. 5C). In contrast to the respiratory system, the digestive system exhibited a different dynamic, with etiologies predominantly detected in the pre-weaning period (before 21 days), with a notable number of rotavirus cases as the single etiology (Fig. 5B). The exception was *Escherichia coli*, which showed an increase in cases among the nursery-age group. Moving to the co-diagnosis dynamics, cases co-diagnosed with *Clostridium spp*. and *Salmonella spp*. had increased frequency in weeks 1 and 5 of age, respectively (Fig. 5D). Unlike the respiratory system, the digestive system had fewer cases throughout the grow-finish age, with the exception of *Lawsonia intracellularis*, which demonstrated a higher number of cases, occurring mainly as a single etiology (Fig. 5B).

### Assessment of insult type on the number of distinct etiologies

Confirmed tissue diagnosis co-diagnosis cases that involved both bacterial and viral agents were higher than co-diagnosis cases involving only bacteria or only viruses (Table 1). Co-diagnosed cases where neither insult type was involved were low, and they were mostly associated with toxicology, parasitic, and nutritional deficiency cases. When including all co-diagnosis cases without categorization based on insult types and anatomic system, it is essential to note the high abundance of PRRSV in the most frequent combinations. PRRSV was present in the 5 more predominant combinations in the whole database, which were: PRRSV + *Streptococcus suis* (5,533), PRRSV + *Pasteurella multocida* (3,085), PRRSV *+ Glaesserella parasuis* (2,899), PRRSV + Influenza A virus (2,560), PRRSV + *Mycoplasma hyorhinis* (2,148).

**Table 1:** Distribution of cases by assessing the presence or absence of bacterial and viral agents, and the most co-diagnosed combination within each group

Presence of Bacterium	Presence of virus	Total co-diagnosed cases	Most co-diagnosed combination
Yes	No	4,070	*Streptococcus suis* + *Glaesserella parasuis*
No	Yes	3, 138	PRRSV + Influenza A virus
Yes	Yes	16, 595	PRRSV + *Streptococcus suis*
No	No	43	Coccidiosis + Cryptosporidiosis
**Total**		23,846	

The COM-Poisson model was selected, as it accommodates over- and underdispersion by allowing the variance to differ from the mean. The COM-Poisson model demonstrated a superior fit, with a substantially lower AIC (58,741) compared to the other models tested. The dispersion was computed as 0.29, which supported the assumption of underdispersion and indicated that the COM-Poisson model was the final model. The RMSE had a low value (0.89), demonstrating a good prediction accuracy for the model.

**Fig. 6:**
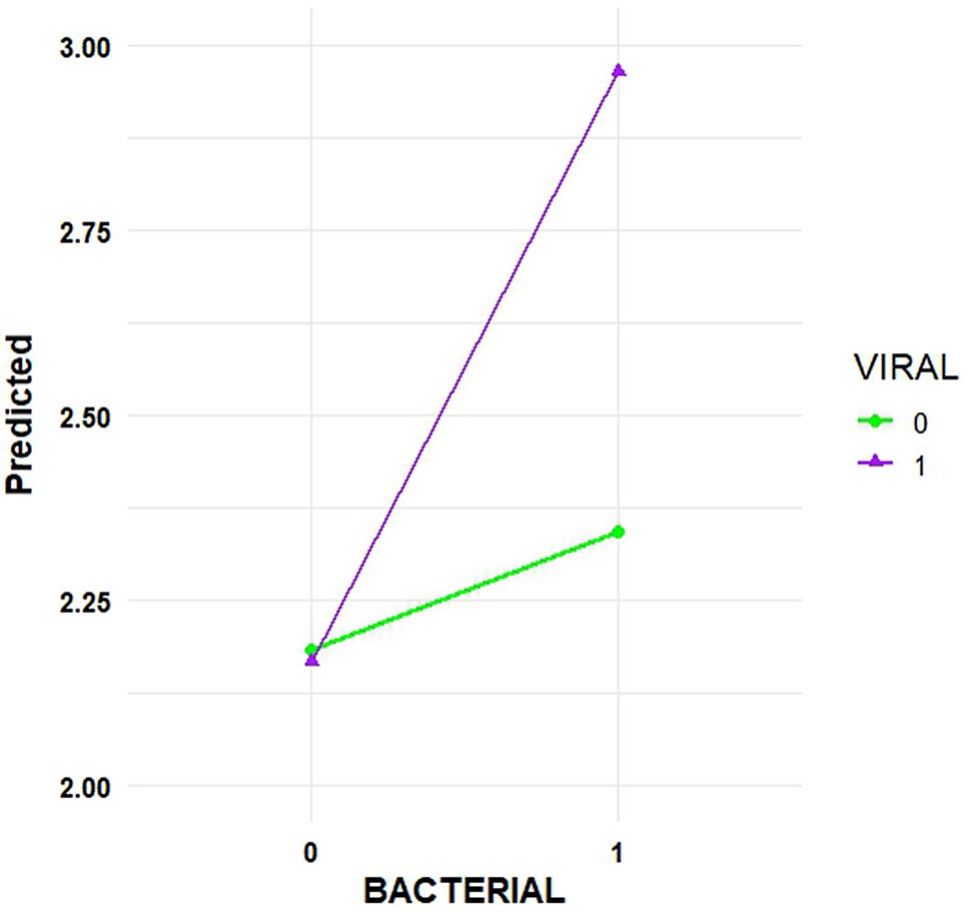
COM-Poisson model estimation of the number of distinct etiologies within a co-diagnosis case (Y-axis) based on the insult type. The green line represents the absence of a virus insult (0), and the purple line represents its presence (1). The X-axis distinguishes cases where a bacterial insult was present (1) or absent (0)

The final COM-Poisson model indicated that co-diagnosis cases involving both bacterial and viral insults were significantly (*p-value* < 0.0001) associated with more distinct etiologies than cases involving neither insult type. Specifically, the incidence rate ratio (IRR) was 2.48 (CI: 1.66; 3.66) higher when both bacterium and virus were diagnosis in the case, and the predicted expected count of distinct etiologies calculated by the model was 2.95 distinct etiologies (Fig. 6). When only a bacterial insult was involved in the co-diagnosis case and no viruses were co-diagnosed, the IRR was 1.27 (CI: 0.86–1.87), which was not statistically significant (*p-value* = 0.84). The same applies to co-diagnosis cases where a virus was involved but no bacteria were involved, with an IRR of 0.96 (CI: 0.65; 1.87), which was also not statistically different (*p-value* = 0.2169). The fact that both confidence intervals of the estimates included the null value one also emphasizes how there was no statistical difference.

When controlling for the presence of the most predominant virus (PRRSV) and bacterium (*Streptococcus suis)*, the changes in the final model estimates were less than 10%. Therefore, we considered that the presence of *Streptococcus suis* or PRRSV was not a cofounder in estimating the number of distinct etiologies in the co-diagnosis cases.

## Discussion

The Dx code system used in this study does not rely solely on pathogen detection results. Instead, it integrates multiple diagnostic inputs, including histopathological lesions, diagnostic assays performed, and case history, before a diagnostician assigns a final code [13]. Therefore, the presence of a pathogen is recorded in the database when it is deemed etiologically relevant and compatible with the observed lesions and clinical context, rather than simply reflecting detection. Thus, the co-diagnosis framework reflected both detection and lesion association, thereby enhancing the epidemiological value of the database by capturing biologically meaningful pathogen interactions.

A consistent seasonal pattern was observed, with a reduction in confirmed tissue diagnoses during the summer months. This aligns with known seasonal dynamics in swine production and disease occurrence [15, 17], where environmental factors, management practices, and biosecurity measures may reduce pathogen transmission or diagnostic submissions [18, 19]. The highest co-diagnosis rate occurred in winter 2021, suggesting that colder months may exacerbate disease risks, particularly for respiratory pathogens [20, 21]. However, when evaluated by the system, the seasonal pattern changes, with digestive co-diagnosis cases increasing in summer, while respiratory cases increase in the fall.

The predominance of co-diagnosis cases in the wean-to-market production phase (86.45%) underscores the vulnerability of pigs in this age group to multifactorial disease processes. This stage involves significant physiological and environmental transitions, possibly predisposing animals to concurrent infections [22, 23]. Furthermore, over time, the low proportion of “unknown” production types suggests improvements in data collection and classification by the VDLs demonstrated by other diagnostic data aggregation research [16, 24]. In addition, the predominance of co-diagnosis cases in Midwest states and North Carolina aligns with the states that have high hog inventory (USDA-NASS, 2025).

Respiratory system involvement was predominant among co-diagnosed cases, with PRRSV, *Streptococcus suis*, or both being the most frequently co-diagnosed pathogens associated with any etiology in respiratory cases. Historically, from 2010 to 2019, PRRSV and IAV were the leading pathogens in respiratory co-diagnosed cases [14]. However, data from ISU-VDL corresponding to 2017–2022 have shown a marked rise in *Streptococcus suis* diagnoses, especially in cases involving bronchopneumonia, meningitis, and endocarditis [25]. The increased number of *Streptococcus suis* co-diagnosis cases likely reflects changes in diagnostic practices, greater recognition of this bacterium as a systemic pathogen [25], and its frequent co-occurrence with viral infections, such as PRRSV and IAV.

The result supports the PRDC concept, where viral and bacterial agents synergistically contribute to disease severity [1, 26, 27], and were present in multiple co-diagnosis cases as described in this study, mainly in the nursery and grow-finish ages. The digestive system exhibited a distinct age-related pattern, with early-life infections predominantly dominated by *Escherichia coli* and rotavirus, and nursery-phase infections involving coccidiosis, PEDV, and *Salmonella spp*. The exception was *Lawsonia intracellularis*, which had more cases in the grow-finish phase after 10 weeks of age. These findings reflect the known epidemiology of enteric diseases and highlight the importance of age-specific diagnostic and management strategies [3, 9, 28].

The COM-Poisson regression model was selected due to evidence of underdispersion in the data, which other models, such as Poisson and negative binomial models, failed to accommodate [29]. The final model revealed that cases involving both bacterial and viral insults were associated with a significantly higher number of distinct etiologies (IRR = 2.48, CI: 1.66–3.66), with a estimated count of 2.95 etiologies per case. This finding highlights the additive or synergistic nature of co-infections involving bacteria and viruses, supporting the hypothesis that cases involving multiple etiologies are more complex and diagnostically challenging [30, 31]. Interestingly, co-diagnosis cases involving only bacterial or viral insults did not show statistically significant increases in the number of distinct etiologies. However, the number of co-diagnosis cases by animal age in a week demonstrated that several bacteria increased the number of cases when associated with other etiologies, providing insight into diagnostic strategies, since bacteria like *Clostridium spp*., *Glaesserella parasuis*, *Pasteurella multocida*, *Streptococcus suis,* and *Salmonella spp.* were more frequent in cases also having other etiologies than in single etiology cases. This pattern suggests that these bacteria often act as secondary or opportunistic pathogens, highlighting the complexity of swine disease cases and the need for comprehensive diagnostic approaches that consider potential co-infections rather than focusing solely on single-agent detection [32].

Additionally, the association between dual insult cases (bacterial and viral) and increased etiological diversity has important implications for swine health management. Diagnostic protocols should be designed to detect multiple pathogens, not only focus on the primary cause, especially in cases with non-specific disease clinical signs [33]. Moreover, the findings highlight the need for integrated surveillance systems to capture co-infection dynamics and inform targeted interventions. The lack of confounding effects from PRRSV and *Streptococcus suis* in the final model indicates that the observed associations are not driven solely by these dominant pathogens.

While the study provides valuable insights, limitations must be acknowledged. First, the use of diagnostic data inherently depends on submission practices, which may vary by region, season, and farm type [34]. Second, multiple etiologies in a case do not necessarily imply simultaneous infection in the same animal, as submissions may include tissues from multiple pigs. Third, despite efforts to standardize diagnostic coding, inter-laboratory and diagnostician variability in interpretation and reporting may still influence the results due to non-standardization methods, as demonstrated in human laboratories [35]. Additionally, the reliance on confirmed tissue diagnoses may limit the scope of pathogen detection, particularly for agents that are often tested through molecular methods without tissue evaluation, such as PEDV and PDCoV, as demonstrated by the number of molecular tests performed by year [36].

This study demonstrates the value of integrating confirmed tissue diagnosis cases across VDLs to analyze co-diagnosis trends in swine. The findings reveal significant associations between dual bacterial and viral insults and increased etiological complexity, with implications for diagnostic strategies and disease management. The study presented a novel cross-laboratory integration of confirmed tissue diagnoses to investigate co-diagnosis patterns in swine across the United States. By aggregating data from two major veterinary diagnostic laboratories, a broader representation of swine health challenges and the utility of standardized diagnostic coding in epidemiological surveillance could be demonstrated. Even though the number of cases from OH-ADDL was reduced compared with ISU-VDL, they came from two states been one of which, West Virginia, was not present in the ISU-VDL dataset (Fig. 2). Adding additional labs increased regional coverage and, consequently, can improve the ability to early detect regional changes, emergence, and re-emergence of animal health threats. Ultimately, the standardized coding framework approach lays a foundation for future application in different livestock species, such as bovine and avian.

The use of a standardized disease diagnosis code to track confirmed tissue disease in animal populations has the significant advantage of increasing sensitivity by utilizing subject matter expertise to interpret diagnostic testing results before assigning a specific disease code. The prospective maintenance of such a database on an animal health monitoring system, such as the Swine Disease Reporting System [14, 16], will allow for the prospective monitoring of endemic pathogen activity. Such data can be scanned by statistical monitoring algorithms for early identification of emerging or re-emerging endemic diseases [15, 17]. Implementing informatics capabilities will reduce the time required for information generation, enabling decision-makers to consider near-real-time decisions for animal production and health interventions, as well as the development of national animal health strategies for current disease burden causes.

In addition, the quantification of disease occurrence in near real-time would enable the prioritization of the most relevant pathogens and the development of animal health interventions tailored to specific objectives, such as the development of immunological or therapeutic solutions. The feasibility of aggregating disease diagnosis data from two labs was demonstrated and can be expanded to incorporate data from other institutions or species. Importantly, these developments were made possible due to the support and need for such information by the U.S. swine industry to inform animal health decisions. Further expansion at both national and international levels is possible and can be accomplished if human willingness and basic infrastructure resources are available for data exchange. The maintenance of data integration prospectively would provide a holistic overview of disease occurrence, enabling continuous refinement and improvements, allowing the advancements in system to generate more information.

## Data Availability

Restrictions apply to access to additional data, and the standard operating procedure (SOP) that was used to aggregate the information, due to the client and VDLs’ confidentiality, and is not publicly available. SOP procedure can be made available upon reasonable request and approval by the SDRS principal investigator (Dr. Giovani Trevisan at trevisan@iastate.edu), (Dr.Daniel Linhares at linhares@iastate.edu), and the VDL directors (Dr. Rodger Main at main@iastate.edu and Melanie Prarat Koscielny at Melanie.Prarat@agri.ohio.gov).
